# A PCA-Based Active Appearance Model for Characterising Modes of Spatiotemporal Variation in Dynamic Facial Behaviours

**DOI:** 10.3389/fpsyg.2022.880548

**Published:** 2022-05-26

**Authors:** David M. Watson, Alan Johnston

**Affiliations:** ^1^School of Psychology, University of Nottingham, Nottingham, United Kingdom; ^2^Department of Psychology, University of York, York, United Kingdom

**Keywords:** dynamic faces, facial caricaturing, ambient faces, computational neuroscience, face perception

## Abstract

Faces carry key personal information about individuals, including cues to their identity, social traits, and emotional state. Much research to date has employed static images of faces taken under tightly controlled conditions yet faces in the real world are dynamic and experienced under ambient conditions. A common approach to studying key dimensions of facial variation is the use of facial caricatures. However, such techniques have again typically relied on static images, and the few examples of dynamic caricatures have relied on animating graphical head models. Here, we present a principal component analysis (PCA)-based active appearance model for capturing patterns of spatiotemporal variation in videos of natural dynamic facial behaviours. We demonstrate how this technique can be applied to generate dynamic anti-caricatures of biological motion patterns in facial behaviours. This technique could be extended to caricaturing other facial dimensions, or to more general analyses of spatiotemporal variations in dynamic faces.

## Introduction

Faces provide a wealth of information about people including their identity (Ellis, 1975), social traits (Oosterhof and Todorov, 2008), and emotional state (Bruce and Young, 1986; Calder and Young, 2005). Faces encountered in the real world are often dynamic and highly variable. Despite this, much research to date has employed static images of faces taken under tightly controlled conditions. Such images risk controlling away potentially important sources of variation, such as within-person variability (Jenkins et al., 2011). Furthermore, both behavioural (Lander and Butcher, 2015) and neurological (O’Toole et al., 2002; O’Toole and Roark, 2010; Bernstein and Yovel, 2015) evidence supports a processing advantage for dynamic over static faces, indicating that dynamic faces convey information that static faces do not.

Facial caricatures present a common method for studying key dimensions of facial variation (Benson and Perrett, 1991b), by either increasing (caricaturing) or decreasing (anti-caricaturing) differences in facial features between an exemplar and reference face, where the reference is typically a neutral or average face. Such methods have been applied to static images to study facial features underlying the perception of identity (Benson and Perrett, 1991a; Blanz et al., 2000; Leopold et al., 2001; Jiang et al., 2006), age (Burt and Perrett, 1995), and emotional expressions (Calder et al., 1997, 2000; Juricevic and Webster, 2012). Caricaturing dynamic faces poses a further challenge as the process must account for both spatial and temporal patterns of variation. Previous approaches have manipulated the magnitude of motion in facial landmarks during simple facial behaviours, using the resulting motion vectors to drive virtual head models (Hill et al., 2005; Furl et al., 2020, 2022). However, such artificial head models lack many of the features present in real faces. A method for dynamically caricaturing facial behaviours in natural videos is therefore still lacking.

In a recent study (Watson et al., 2020), we developed a paradigm for eliciting dynamic and natural facial behaviours by video recording subjects while they delivered short sentences conveying either good or bad news. Speech patterns such as these provide a key source of non-rigid motion in the face. Using a principal component analysis (PCA)-based active appearance model, along with machine learning, we were able to reconstruct a behaviourally interpretable dimension of emotional valance from the facial behaviours. This technique considered motion information in the sense that it included changes in shape and texture over time. However, it did not consider the temporal structure of this information as each frame was simply represented as an independent sample within the model, and the order of the frames was ignored. It thus remains unclear whether such a model is able to capture more nuanced patterns of temporal variation.

Here we present an alternative application of our previous methods (Watson et al., 2020) that aims to model patterns of both spatial and temporal variation within natural facial behaviours. We demonstrate how this technique can be used to create dynamic anti-caricatures of biological motion patterns by morphing between an exemplar and an average timeseries of facial behaviours evoked during speech movements. As before, we initially use a PCA-based active appearance model to capture modes of spatial variation in the face over time. We then use an established dynamic time warping algorithm to align the PCA timeseries over clips. Finally, we present a novel method for capturing patterns of temporal variation by submitting the PCA timeseries to a further second-order PCA. This second-order PCA space represents deviations between exemplar clips and the average first-order PCA timeseries. Weighting and then back-projecting samples from this space yields anti-caricatured videos that vary in terms of their spatial and temporal deviations from the average timeseries.

## Methods

The datasets and some of the methods presented here have previously been described in Watson et al. (2020).

### Recordings

Three subjects (two females, one male, and age range 26–42) were video recorded. The study was approved by the Ethics Committee of the School of Psychology at the University of Nottingham (Ethics approval number: 717) and conducted in accordance with the guidelines and regulations of this Committee and the Declaration of Helsinki. All subjects provided informed written consent to take part in the study and for their likeness to be used in publication.

Subjects were recorded against a uniform visual background in an anechoic chamber. Recordings were made with a Sony HXR-NX5U NXCAM camera connected to an Atomos Ninja-2 recorder that recorded videos in Apple ProRes RAW format. Videos were acquired at a resolution of 1,920 × 1,080 pixels and at 25 fps with a 6.67 ms exposure. Audio was recorded at a 48 kHz sampling rate. Videos were then encoded using MPEG-4 lossless compression prior to further processing.

Each subject delivered multiple repeats of 20 unique phrases, each conveying either good or bad news (10 unique phrases within each type). A list of the phrases is provided in Supplementary Table 1. Subjects 1 and 2 performed 15 repeats of each phrase (300 total), and Subject 3 performed 16 repeats (320 total). Subjects were not told to pose any specific expressions or behaviours; instead, they were instructed to simply deliver the phrases in whatever manner felt most natural to them. While delivering the phrases, subjects viewed silent videos of putative recipients presented on a teleprompter directly in front of the camera. Recipient videos showed video-conference style calls obtained from YouTube and helped give subjects the impression of having a person listen to them while they delivered their phrases.

### Video Pre-processing

Each phrase repeat was then clipped to just the common prefix portion of each phrase (“Good news …” or “I’m sorry to say …”), excluding the later variable suffix portions (e.g., “… the operation went well!” or “… we’re going to have to let you go”). The Google Cloud Speech-to-Text algorithm^1^ was used to generate timestamps for each word in each phrase, which were then used to define onsets and offsets for each prefix portion. Onsets were adjusted 200 ms before the first word onset so as to include facial movements commencing immediately prior to the vocalisation. Manual corrections were applied where necessary. Clips varied in duration because the length of each vocalisation could differ over repeats.

Each clip was then cropped to a square region around the face. A Haar cascade face-detection algorithm implemented in OpenCV^2^ extracted the position of the face within the scene on each frame. A square bounding box was then defined around the average face position, allowing for a small border around the face. This ensured the face was placed approximately centrally within the scene. Each clip was then down-sampled to a resolution of 128 × 128 pixels via an anti-aliasing filter.

### Multi-Channel Gradient Model and First-Order Principal Component Analysis

**FIGURE 1:**
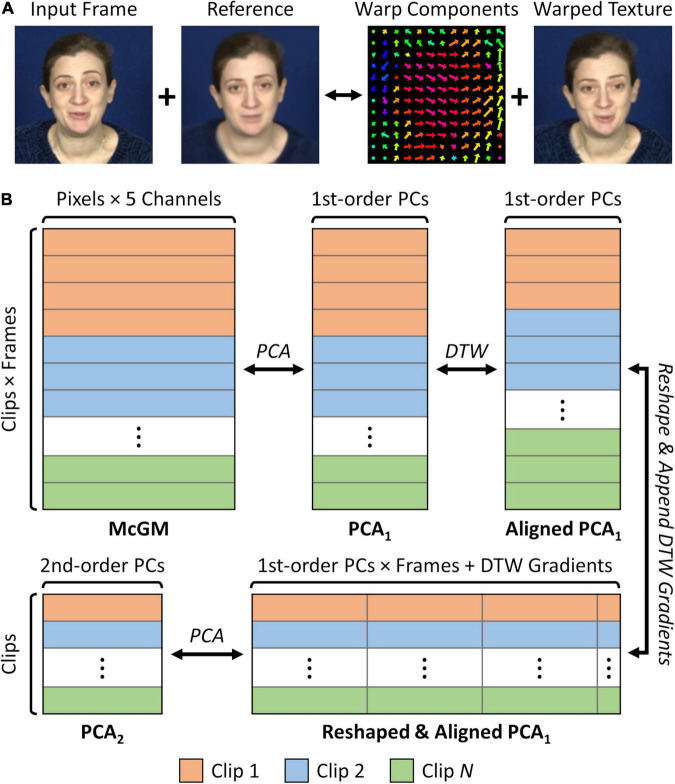
Schematic illustration of processing pipeline. **(A)** Multi-channel Gradient Model (McGM) registration process. Each input frame is warped to an average reference image, producing a 5-channel image comprising the *x*- and *y*-warp components plus the RGB warped textures. **(B)** Principal Component Analysis (PCA) pipeline. McGM outputs are vectorised such that each frame is represented as a sample in a high dimensional space. This space is reduced via lossless PCA, yielding the PCA_1_ space. Clips are temporally aligned by interpolating PCA_1_ scores between frames based on dynamic time warping (DTW) of the audio streams. Aligned PCA_1_ scores are vectorised within each clip and combined with the dynamic time warping gradients, such that each clip is represented as a sample in a new high dimensional space. This space is then reduced by a second lossless PCA, yielding the PCA_2_ space. The pipeline is fully reversible, such that samples at any stage can be back-projected to the image space.

An overview of the remaining processing pipeline is illustrated in Figure 1. We employed a two-frame version of the Multi-channel Gradient Model (McGM; Johnston et al., 1992, 1999; Cowe, 2003) to capture shape and texture changes in the face over frames. This model has previously been shown to capture key dimensions of facial variation including gender (Griffin et al., 2011), speech movements (Scholes et al., 2020), and emotional valence (Watson et al., 2020), and can identify critical facial features for image reconstruction (Berisha et al., 2010). For each frame within the cropped and down-sampled clips, a warp vector field was calculated to register the frame to a standard reference image. The reference image was initially defined by an individual frame extracted from one of the recordings but was then replaced with the average of all textures after warping. This process was iterated three times, recalculating the warps and replacing the reference image with the average warped textures each time: this provided a more standardised final reference image. For each frame, the McGM yields a 5-channel image comprising the *x*- and *y*-direction warp components needed to warp the original image to the final average reference image, plus a “shape-free” version of the RGB textures after warping to the reference. These images were then vectorised and stacked over frames and clips, such that each frame in each clip is represented as an independent sample within an 81,920-dimensional (128 × 128 × 5) feature space defined by the pixels of the McGM images. Each clip is therefore represented by a high-dimensional multivariate timeseries within the McGM space. Note that the use of the McGM here for image registration (Cowe, 2003) differs from some previous applications that instead used the same model to measure local image velocities (Johnston et al., 1992, 1999).

Samples within the McGM space were then split between the phrase-types (“Good news” and “I’m sorry to say”), and each phrase-type was processed separately thereafter. The dimensionality of the McGM space was reduced via principal component analysis (PCA). All available components were retained (one fewer than the total number of frames over all clips within the given phrase-type). Because the number of samples is less than the number of McGM features, this allows a lossless PCA where 100% of variance remains explained while still reducing the dimensionality. Whereas a lossy PCA can reduce the dimensionality further, the lossless PCA was chosen to best preserve the fidelity of individual clips. This produced the first-order PCA (PCA_1_) space, in which samples are given by the frames over all the clips (within the given phrase-type), and dimensions are given by the first-order principal components. Each clip is thus represented by a multivariate timeseries within the PCA_1_ space. The first-order principal components encode modes of common spatial variation amongst the McGM image pixels, but do not consider the temporal order of this information.

### Temporal Alignment

Next, we temporally aligned the PCA_1_ timeseries over the clips within each phrase-type independently. Although all clips were cut to the initial prefix portion of each phrase, the onset of each vocalisation won’t necessarily occur at the same time point within each clip, and the duration of each vocalisation may vary over repeats. We based the temporal alignment on the audio streams as they provide a precise measure of the temporal evolution of each vocalisation and show a good correspondence over repeats.

Audio streams were averaged over stereophonic channels to produce a single monophonic audio signal for each clip. As we are primarily interested in aligning modulations in the audio amplitudes, we applied a Hilbert transform to extract the amplitude envelopes from the audio signals. The original cuttings of each clip include a brief period prior to the onset of the first word to capture any facial movements commencing immediately prior to the start of the vocalisation. However, these periods are largely silent and so lack any consistent amplitude modulations on which to base a temporal alignment. We therefore re-cut the onset of each audio stream to lie closer to the onset of the actual vocalisation. This was done by identifying the timepoint of the initial rise in audio amplitude in the amplitude envelope. This timepoint was then adjusted to 80 ms (two video frames) prior to this to allow a small margin of error prior to the audio onset, and then rounded to the timepoint of the nearest video frame onset (i.e., to the nearest 40 ms). Manual corrections were applied where necessary.

**FIGURE 2:**
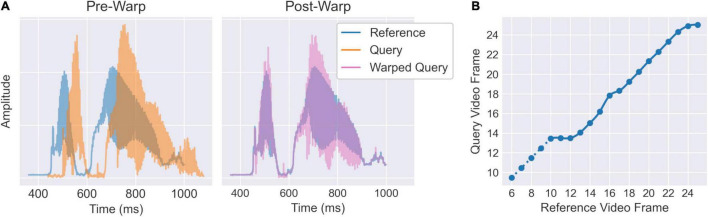
Illustration of temporal alignment procedure for an example “Good news” clip. **(A)** Audio amplitude envelopes for each clip are aligned to an average reference envelope via dynamic time warping. **(B)** The resulting warping curve is down-sampled to the video sampling rate such that it can be applied to the PCA_1_ timeseries. The interpolated curve is extended at a 45° angle for 4 video frames prior to the audio onset (dotted line) so that facial movements commencing shortly before the vocalisation are also included.

The re-cut audio streams were then temporally aligned using a dynamic time warping (DTW) algorithm implemented using the *dtw-python* package (Giorgino, 2009; Tormene et al., 2009)^3^. For purposes of computational tractability, the audio amplitude envelopes were down-sampled by a factor of 10 (yielding an effective sampling rate of 4.8 kHz) using scipy’s *decimate* function. The down-sampled envelopes for each clip were then aligned to a common reference envelope (Figure 2A). The reference was initially selected as the individual clip closest matching the median duration over all clips. However, to provide a more standardised reference, the reference envelope was then replaced with the average envelope over all clips after temporal alignment, and the DTW was recomputed for the new reference. This process was iterated three times, updating the average reference envelope each time, to allow the reference to stabilise. To minimise extraneous effects of global amplitude differences irrelevant to the temporal alignment, all envelopes (including the reference) were rescaled to have an L^2^-norm equal to one on every iteration. The DTW was computed using an asymmetric step pattern and allowed open beginnings and ends so that neither the first nor last sample need be matched exactly.

The resulting warping curves can be used to align the audio streams. To align the video frames, the audio warping curves were down-sampled to the resolution of the video sampling rate (25 fps) via linear interpolation (Figure 2B). The audio alignment excludes the period before the vocalisation onset; however, some facial movements may still occur during this period. We therefore extended the video warping curves at a 45° angle (i.e., matching clip and reference frames one-for-one) for a further four video frames (160 ms) prior to the vocalisation onset. Although this may only achieve a moderately accurate temporal alignment, it is nevertheless preferable to include the visual information from this initial period than to exclude it. The final video warping curves were then used to apply a linear interpolation between frames of the PCA_1_ timeseries for each clip. Following this, each clip is still represented by a multivariate timeseries in the PCA_1_ space, however, all timeseries will now be the same length and should be aligned in time (within each phrase-type).

### Second-Order Principal Component Analysis and Caricaturing

The temporally aligned PCA_1_ scores were vectorised within each clip. In addition, the gradients of the video time warping curve were appended to the end of each vector: this allows incorporating information about the temporal scale of the behaviours and permits the DTW curve to be reconstructed from the second-order PCA space. This generated a new high-dimensional feature space in which each clip is represented as an independent sample, and where the dimensions comprise the concatenation of first-order principal components over video frames plus the DTW gradients. The dimensionality of this space was then reduced by a further lossless PCA. As before, all available components were retained (one fewer than the number of clips), such that 100% of variance remained explained after the dimensionality reduction. This produced the second-order PCA (PCA_2_) space, in which each clip is a sample and the dimensions are given by the second-order principal components. Components within this space can reflect interactions between first-order principal components and timepoints and can therefore encode both spatial and temporal modes of facial variation. Whereas points within the PCA_1_ space represent individual images, points within the PCA_2_ space represent full temporal sequences. The origin of the PCA_2_ space represents the PCA_1_ timeseries averaged over clips, and individual samples are represented in terms of their spatiotemporal deviations from this average timeseries.

To produce dynamic anti-caricatures, we weighted the loading of a given sample/clip within the PCA_2_ space. A weighting of zero will reduce the representation to the origin, thereby reproducing the average PCA_1_ timeseries. A weighting of one will return a representation of the original clip. Intermediate weightings will yield varying levels of anti-caricature, weightings greater than one will yield caricatures, and negative weightings will yield anti-faces. For a given weighting, the resulting PCA_2_ sample was back-projected to the reshaped PCA_1_ space. This produced a reconstruction of the vectorised PCA_1_ scores plus the DTW gradients. The reconstructed vectorised PCA_1_ scores were then “un-vectorised” to return the sample to the temporally aligned PCA_1_ space (with frames as samples and first-order principal components as dimensions). Meanwhile, the reconstructed DTW gradients were used to generate a time warping curve, which was in turn then used to apply a linear interpolation to the “un-vectorised” reconstructed PCA_1_ scores that returned them to the original timescale. From here, the reconstructed PCA_1_ scores were back-transformed to the McGM space. Finally, the reconstructed McGM warp components were inverted to spatially unwarp the reconstructed textures back to the image space. To aid visualisation, the visual contrast of the images was enhanced via unsharp masking. The complete back-projection of a given point from within the PCA_2_ space therefore yields a full video animation within the image space.

### Perceptual Ratings

We conducted a behavioural experiment to quantify the effect of the caricaturing on human perception of biological motion in the videos. Ten participants took part in the experiment (three male, seven female, and age range 23–36). The study was approved by the Ethics Committee of the School of Psychology at the University of Nottingham (Ethics approval number: F1249) and conducted in accordance with the guidelines and regulations of this Committee and the Declaration of Helsinki. Participants provided informed consent via an electronic form prior to participation. The experiment was run online using PsychoPy and Pavlovia (Peirce et al., 2019)^4^. To avoid confusion, for the analysis of the behavioural data we refer to the participants in this experiment as “raters” and the participants in the original video recordings as “recording subjects.”

Raters were shown video clips across 5 levels of anti-caricaturing (0, 0.25, 0.5, 0.75, and 1) for the “I’m sorry to say” phrases. We avoided using weightings outside the zero to one range (caricatures and anti-faces) as these are more prone to image distortions that could confound the task. We also omitted the “Good news” phrases as these typically have relatively short durations which would make the task unduly challenging. Each rater was shown a 10% subset of clips across all three recording subjects (15 unique clips for S1 and S2, 16 unique clips for S3), such that ratings were provided for all clips across the 10 raters. Across the 5 caricaturing levels, each rater therefore completed 230 trials. The trials were split into three blocks with each recording subject presented continuously throughout a block; this was done to aid raters in generating an internal standard of each recording subject’s range of facial behaviours. The order of blocks was randomised for each rater. An additional shorter practice block was included at the start of the experiment, comprising the 5 anti-caricaturing levels for an example clip from each recording subject (15 trials total). To avoid priming responses, practice clips were selected from a different subset of clips to the main trials.

Raters were informed that they would view a series of silent videos showing people saying a short phrase, and that the people might appear livelier and more dynamic in some videos, and less so in others. A more precise definition of dynamicity was deliberately omitted so as to encourage raters to form their own interpretation. On each trial, raters viewed the video clip and were then asked to rate it for how “dynamic” the person appeared to be. Raters made their responses on a 5-point Likert-scale with the labels: “Not at all,” “Not much,” “A bit,” “Fairly,” and “Very.” The responses were entered into a mixed effects ordinal logistic regression implemented using the *ordinal* package in R (Christensen, 2019)^5^. The caricaturing level (0, 0.25, 0.5, 0.75, and 1) was entered as the predictor variable, while the dummy coded ratings (1–5) were entered as the outcome variable. Variable intercepts were allowed over raters (R1–R10) and recording subjects (S1–S3); a more complicated model allowing variable slopes failed to converge. The slope parameter (β_1_) represents the log odds of giving a higher versus lower dynamicity rating given a one unit increase in the caricaturing level. If the slope is significantly greater than zero, this would indicate that increasing the caricature level leads to a significant increase in the likelihood of raters providing a higher rather than lower dynamicity rating. We applied an alpha criterion of 0.05 for determining statistical significance.

## Results

**FIGURE 3:**
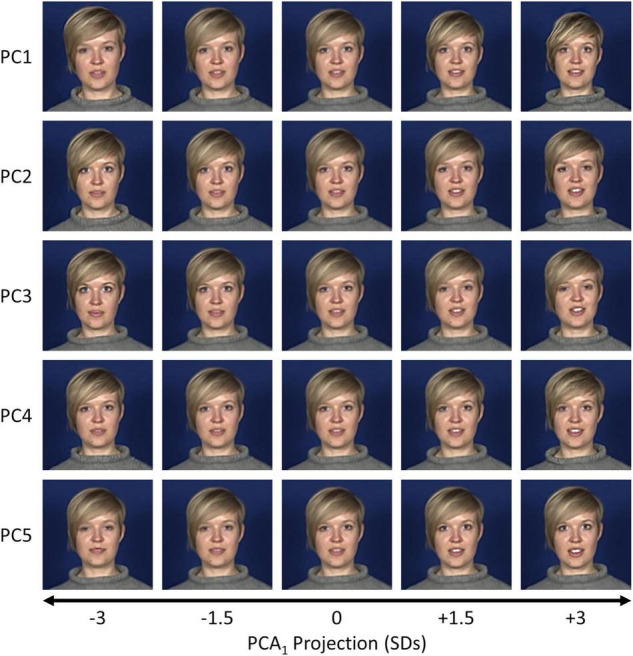
Visualisations of the first five components from the PCA_1_ space for an example dataset (S3, “Good news”). Samples were selected at positions between ± 3 standard deviations along a given principal component axis and back-projected to the image space.

Three subjects were video recorded while delivering a series of phrases conveying either positive (“Good news”) or negative (“I’m sorry to say”) news, eliciting dynamic and natural facial behaviours in the form of speech patterns. A two-frame version of the Multi-channel Gradient Model (McGM; Johnston et al., 1992, 1999; Cowe, 2003) was used to register the facial textures in each frame to a common average reference frame (Figure 1A). Each frame is then represented by a 5-channel image comprising the *x*- and *y*-direction warp components plus a “shape-free” version of the RGB textures. The remaining processing pipeline is illustrated in Figure 1B. This pipeline is applied within each phrase-type (“Good news” and “I’m sorry to say”) and for each subject independently. First, the McGM outputs were vectorised such that each frame is represented as a sample within a high-dimensional feature space. We then reduced the dimensionality of this space using a lossless Principal Components Analysis (PCA), retaining all available components so that 100% of the variance remains explained. The resulting feature space is hereafter referred to as the first-order PCA (PCA_1_) space. Components within this space encode principal modes of spatial variation in facial shape and texture over frames (Turk and Pentland, 1991), but do not consider the temporal order of such changes. Visualisations of the features encoded by the early principal components are shown for an example dataset in Figure 3, and animations for all datasets are shown in Supplementary Video 1. The first principal component typically encodes global changes over clips such as seating position or a change in clothing; exaggerations of this component often induce distortions in the image. Later components encode more salient modes of facial variation, including both rigid changes in the head position and non-rigid changes in the internal facial features such as in the shape of the mouth or opening of the eyes.

**FIGURE 4:**
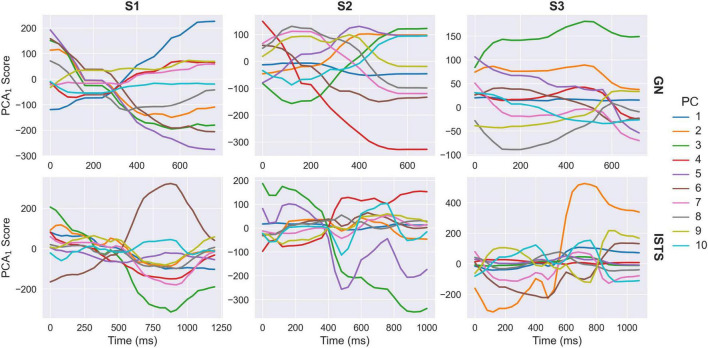
PCA_1_ timeseries averaged over clips following temporal alignment. Plots illustrate cross-sections through the first ten principal components for each subject and phrase-type (GN, “Good news”; ISTS, “I’m sorry to say”).

At this point, each clip is represented by a multivariate timeseries within the PCA_1_ space. However, these timeseries are not temporally aligned over clips and may be different durations. To capture common patterns of temporal variation, it is therefore necessary to temporally align the PCA_1_ timeseries. We based the alignment on the audio streams within each clip as these provide a precise index for the temporal evolution of each vocalisation. We used a dynamic time warping (DTW) algorithm to temporally align the audio envelopes, then down-sampled the resulting warping curves to the video sampling rate and used these to interpolate the PCA_1_ timeseries between frames in each clip (Figure 2). Following time warping, all PCA_1_ timeseries are identical in duration and temporally aligned over clips. Figure 4 illustrates cross-sections through the first ten aligned PCA_1_ timeseries averaged over clips. Clear modulations are present in each component timeseries, indicating both that common patterns of temporal variation are present in the PCA_1_ scores and that the time warping procedure was successful in aligning these. This can be further illustrated by back-projecting the average PCA_1_ timeseries to the image space. The resulting animations (Supplementary Video 2) maintain clear depictions of the phrases (“Good news” or “I’m sorry to say”) being spoken.

We next extracted common patterns of temporal variation from the PCA_1_ timeseries. The temporally aligned PCA_1_ scores were vectorised within each clip independently and concatenated together with the DTW gradients for each clip. Including the DTW gradients allows incorporating information about the temporal scale of the behaviours and ensures that back-projections through this space include the necessary information to “unwarp” the corresponding PCA_1_ timeseries back to their original timescale. This produces a new high-dimensional feature space, in which each clip is represented as a sample, and where the dimensions are defined by the combination of PCA_1_ components and timepoints plus the DTW gradients. This space was then reduced via a further lossless PCA, again retaining all available components such that 100% of variance remains explained: this yields the second-order PCA (PCA_2_) space. Components in the PCA_1_ space capture principal modes of spatial variation in the faces, but without regard to the temporal order of those changes. By contrast, components in the PCA_2_ space capture patterns of temporal variation amongst the first-order principal components. Whereas each point within the PCA_1_ space represents an individual image, each point within the PCA_2_ space represents a full temporal trajectory and can be visualised as a video if back-projected to the image space. The origin point of the PCA_2_ space represents the average PCA_1_ timeseries (Figure 4 and Supplementary Video 2), and individual clips are represented within the PCA_2_ space in terms of their spatiotemporal deviations from this average timeseries.

**FIGURE 5:**
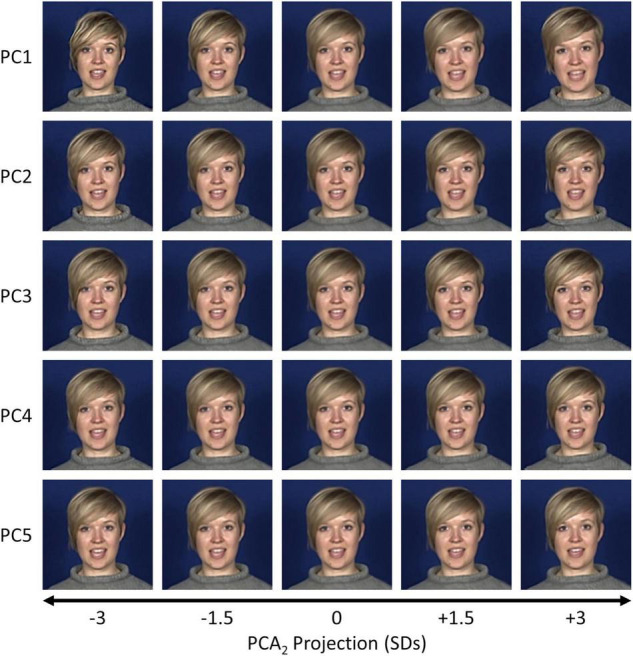
Visualisations of the final frame in the sequence for the first five components from the PCA_2_ space for an example dataset (S3, “I’m sorry to say”). Samples were selected at positions between ± 3 standard deviations along a given principal component axis and back-projected to the image space.

To visualise this more clearly, we back-projected samples at varying positions along the first five components of the PCA_2_ space. Still images from the final frame in each sequence for an example dataset (Subject 3, “I’m sorry to say”) are shown in Figure 5, and animations of the full sequences are shown in Supplementary Videos 3–8. Similar to the PCA_1_ space, early components encode global shape changes and exaggerating them often causes distortions in the image. Patterns of facial variation encoded in later components tend to be more subtle than those observed in the PCA_1_ space (cf. Figure 3) but are still evident. For example, in Subject 3’s “I’m sorry to say” dataset, PCs 1 through 4 include differences in the head tilt, while PC 5 modulates the vertical head and jaw position.

**FIGURE 6:**
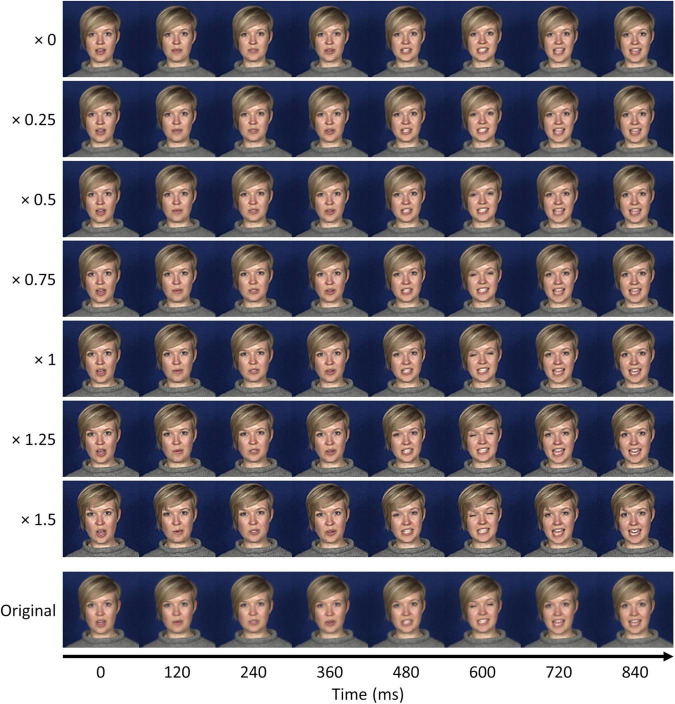
Still frames from dynamic anti-caricatures and caricatures of an example clip (S3, “I’m sorry to say”). Weighting by zero yields the average video sequence, and weighting by one reconstructs the original clip. Intermediate weightings yield varying levels of anti-caricature, while weightings greater than one yield caricatures. The bottom row illustrates frames from the original clip matched to approximately the same timepoints.

Dynamic anti-caricatures and caricatures (Leopold et al., 2001) of individual clips can be produced by multiplying a given clip’s loadings within the PCA_2_ space and back-projecting to the image space. A weighting of zero will reduce to the origin of the PCA_2_ space and hence reproduce the average PCA_1_ timeseries. A weighting of one will reproduce the timeseries of the original clip. Intermediate weightings will produce varying levels of anti-caricature between the individual and average timeseries. Weightings greater than one will produce caricatures that exaggerate the deviations between the individual and average timeseries (Benson and Perrett, 1991b), while negative weightings will produce anti-faces that invert the deviations (Leopold et al., 2001). Back-projecting a weighted sample to the image space produces a dynamic (anti-)caricatured video. Figure 6 shows still images from anti-caricatured and caricatured sequences for an example clip from Subject 3’s “I’m sorry to say” data. Animations for example clips from other datasets are shown in Supplementary Videos 9–14, including anti-faces, anti-caricatures, and caricatures. The caricaturing process can modulate the intensity of multiple idiosyncratic behaviours within each clip, such as the head orientation, head movements, blinks, and mouth movements. Because the original behaviours all occurred across relatively similar timescales, the caricaturing effects here are most salient for spatial features. Nevertheless, modulations of temporal features are also evident; for instance, the caricaturing also alters the duration of sequences that are shorter or longer than the average sequence. Weightings outside of the zero to one range (caricatures and anti-faces) are prone to introducing distortions into the image, especially in the case of the anti-faces. This is because the McGM features represent the facial motion in terms of changes in shape and texture over time; modulating these features outside the normal range will therefore exaggerate shape as well as temporal deviations, leading to shape distortions. Consequently, this technique may be best suited to producing anti-caricatures, using weightings within the zero to one range.

**FIGURE 7:**
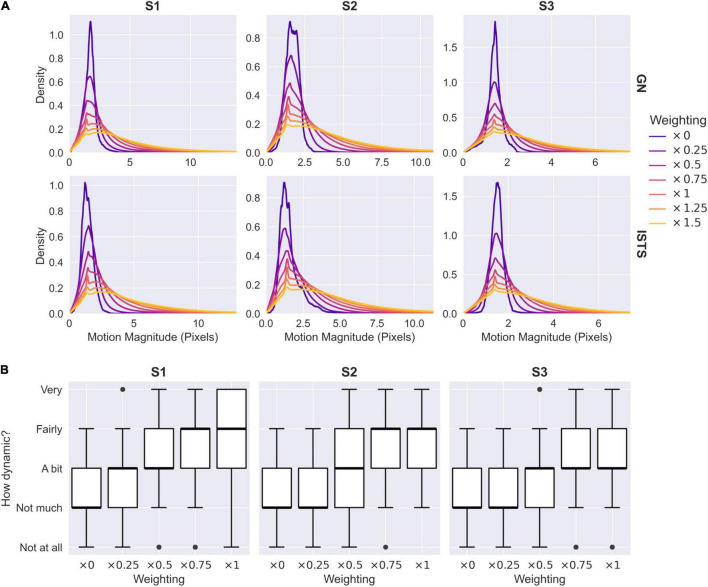
Objective and perceptual measures of caricaturing. **(A)** Magnitudes of visual motion across varying levels of caricature and anti-caricature. Weighted samples are back-projected from the PCA_2_ to the McGM space, and the magnitudes of the *x*- and *y*-warp components are extracted. Kernel density estimates illustrate distributions of magnitudes over all frames in all clips. GN, “Good news”; ISTS, “I’m sorry to say.” **(B)** Perceptual ratings of dynamicity across varying levels of anti-caricature for the “I’m sorry to say” phrases. Boxplots illustrate medians, upper and lower quartiles, and data ranges excluding outliers.

To quantify the relationship between the caricaturing process and the extent of biological motion, we conducted two further analyses. First, we obtained an objective measure of the degree of motion in each video sequence by calculating the magnitude of the vectors in the *x*- and *y*-warp components of the McGM feature space. These represent the magnitude of deviation between each frame and the original reference image (Figure 1A), such that larger values indicate a greater degree of movement over frames. Distributions of motion magnitudes over frames are illustrated in Figure 7A for varying anti-caricature and caricature levels. As the level of caricaturing increases the distributions become increasingly broad and biased toward larger values, indicating greater magnitudes of motion in the clips. Secondly, we obtained perceptual ratings from 10 naive observers for the dynamicity of each clip across anti-caricature levels of the “I’m sorry to say” phrases. Summaries of the ratings are illustrated in Figure 7B: as the level of caricaturing increased so too did the dynamicity ratings. This was confirmed with a mixed-effects ordinal logistic regression, which revealed that increasing the caricature level significantly increased the likelihood of providing a higher dynamicity rating [β_1_ = 3.62, exp(β_1_) = 37.20, *z* = 27.09, *p* < 0.001]. Thus, both objective and perceptual measures indicated that the caricaturing process successfully modulated the degree of biological motion.

## Discussion

In this study, we present a novel method for capturing spatiotemporal patterns of biological motion in dynamic facial behaviours and demonstrate how this can be used to create dynamic anti-caricatures of those behaviours. This technique extends existing spatial caricaturing methods by allowing manipulation of both spatial and temporal features. A PCA-based active appearance model is first used to capture dimensions of spatial variation. Following temporal alignment of the PCA timeseries, the scores are then submitted to a second-order PCA that further encodes spatiotemporal variations amongst facial behaviours. Each of these PCA spaces embodied all gestured behaviours, including both rigid and non-rigid modes of facial variation. Weighting a given sample within the second-order PCA space yields dynamic (anti-)caricatures of that sequence relative to the average first-order PCA timeseries. Both objective and behavioural measurements confirmed this technique modulated the degree of biological motion in the facial behaviours.

Facial caricatures offer an important tool for studying face perception by allowing parametric manipulation of key facial dimensions that would be difficult or impossible for a person to pose naturally. Caricaturing manipulations predict behavioural ratings of corresponding facial features (Benson and Perrett, 1991a; Burt and Perrett, 1995; Calder et al., 1997, 2000; Blanz et al., 2000; Furl et al., 2022), produce perceptual adaptation effects (Leopold et al., 2001; Jiang et al., 2006; Juricevic and Webster, 2012), and predict neural responses in face-selective regions (Furl et al., 2020). Our approach extends existing spatial caricaturing techniques by providing a method for generating dynamic anti-caricatures from natural facial behaviours, thereby allowing investigation of both spatial and temporal features underlying face perception. The dimensions manipulated by the caricaturing will depend on the choice of reference, determined by the average of the clips in the first-order PCA space. In our demonstration, this was an average of all clips within a given phrase type (“Good news” or “I’m sorry to say”), and thus the caricaturing manipulated idiosyncratic behaviours and biological motion patterns in each clip relative to this average. We derived dynamic facial behaviours from speech patterns: these provide a good target case as they represent a key source of non-rigid motion in the face, and also provide a degree of regularity that is helpful for forming a temporal average. Nevertheless, the technique could be applied to any pattern of facial movements provided that some temporal average (or other reference) can be formed for such movements. Such behaviours could, for instance, include poses of emotional expressions or head turns. Dynamic caricatures of facial behaviours could be applied to study recognition of those behaviours (Furl et al., 2020, 2022), similar to how static caricatures have been used to characterise recognition of facial identity (Leopold et al., 2001). Indeed, our own behavioural results confirmed that observers’ perception of facial dynamicity increased with increasing levels of caricaturing. Furthermore, this technique could be extended to manipulating and characterising other facial characteristics in dynamic videos, such as emotion (Calder et al., 1997), identity (Benson and Perrett, 1991a), or age (Burt and Perrett, 1995).

A key component of this process is that the first-order PCA timeseries should be temporally aligned before conducting the second-order PCA. We used a dynamic time warping process based on the audio streams as this offered a precise measure of the vocalisation timings, but similar methods could be applied to other time-varying metrics such as the position of key facial landmarks (Hill et al., 2005; Furl et al., 2020). We included the time warping gradients along with the vectorised PCA_1_ timeseries as inputs to the second-order PCA - this served a theoretical purpose by including information about the temporal scale of the original facial behaviours, as well as a practical purpose by ensuring that back-projections from the PCA_2_ space would reconstruct the gradients needed to “unwarp” the corresponding reconstructed PCA_1_ timeseries back to its original timescale. It is important to note that a poor temporal alignment amongst clips will likely cause modulations in the first-order PCA timeseries to cancel and average out over clips, resulting in a reference timeseries that corresponds to a largely static face. Dynamic caricatures generated relative to a static reference may include undesirable properties, such as a modulation of global motion rather than of idiosyncratic facial behaviours. For instance, Hill et al. (2005) note that caricaturing a dynamic negative expression relative to a static neutral reference would have the effect of increasing the overall dynamicity of the expression, yet we might expect an expression would actually become less dynamic with increasingly negative valence.

Here, we obtained recordings from a relatively small number of subjects, but with each subject performing many repeats of each phrase. This design allowed us to build PCA-based active appearance models that optimally characterised facial behaviours within each subject individually. Future applications of these techniques might additionally explore spatiotemporal variation between subjects or between different facial behaviours. For instance, PCA-based spatial caricaturing techniques have previously been used to morph between individuals varying in features such as gender (Griffin et al., 2011) and perceived political affiliation (Roberts et al., 2011). A more variable stimulus set comprising a wider variety of individuals or facial behaviours may prove more beneficial for such investigations.

Previous facial caricaturing methods have typically relied on manipulating the difference between an exemplar and reference face in terms of pre-defined facial landmarks. By contrast, the McGM employed here captures dynamic changes in shape and texture at the pixel-level (Johnston et al., 1992, 1999; Cowe, 2003). This allows our approach to advance on previous dynamic caricaturing methods by permitting manipulation of the original video textures instead of driving a virtual avatar. Consequently, our technique can represent finer and more nuanced changes in the faces, which would potentially be lost if only sampling sparse facial landmarks. Furthermore, our approach can capture changes in the texture and shape from shading that can be challenging to represent accurately in a virtual avatar. Nevertheless, our technique can be more prone to image distortions, especially outside the anti-caricature range (i.e., generating caricatures or anti-faces), which are less prevalent in virtual avatars. Manipulating the video textures may also modulate other incidental image properties; for instance, visual contrast was generally reduced for lower anti-caricaturing levels due to the averaging process. Visual contrast can influence the perception of various facial features including attractiveness (Pallak, 1983), age (Porcheron et al., 2017), emotional expression (Webb et al., 2020), and first impressions (Sato et al., 2008). The facial dynamicity ratings provided in our own behavioural experiment may potentially have been influenced by changes in visual contrast over varying levels of anti-caricature, although these changes would not be expected to influence facial motion directly, and subjective accounts of varying dynamicity are consistent with our objective measurements of visual motion magnitudes. Future applications of this technique may therefore consider whether further control or normalisation of such image features would be beneficial. Thus, the advantages and disadvantages of each approach may be best considered relative to the use case. Other methods based on temporal filtering have also been proposed for exaggerating motion in dynamic scenes (Wu et al., 2012), however, these produce a general increase of all motion within the scene, while our approach more specifically targets dynamic facial behaviours.

Weighting a sample in the second-order PCA space between zero and one allows generating varying levels of anti-caricature. Multiplication by values greater than one can create active caricatures, in which dynamic behaviours are exaggerated beyond the level present in the exemplar clip. Equally, multiplication by negative values can generate “anti-face” (anti-)caricatures, in which the encoded facial behaviours are inverted (Blanz et al., 2000; Leopold et al., 2001; Jiang et al., 2006; Juricevic and Webster, 2012; Furl et al., 2020, 2022). In our approach, however, multiplication outside the zero to one range (Supplementary Videos 9–14) tended to produce distortions in the image, particularly in the case of the anti-faces. The McGM features represent the motion of the face in terms of changes in shape and texture over frames. Modulation of these features outside the normal range will therefore exaggerate shape as well as temporal deviations, leading to shape distortions. At present, our technique may be best suited for generating dynamic anti-caricatures. Existing caricaturing techniques, such as those driving virtual avatars, may be more appropriate for generating more extreme caricatures or anti-faces depending on the use case.

Here we demonstrate the utility of our method for deriving dynamic anti-caricatures, however, it could be extended for many other purposes. First-order PCA face spaces have been used to classify and extract features underpinning emotional expressions (Calder et al., 2000; Watson et al., 2020) and facial identity (Kramer et al., 2017). They have also been used to generate predictive models of behavioural (Hancock et al., 1996, 1998) and neural (Chang and Tsao, 2017) representations of faces. The second-order PCA approach described here offers the opportunity to extend such investigations to include both spatial and temporal modes of facial variation. While the second-order PCA aims to identify variation along orthogonal linear components, other decomposition techniques may also be able to utilise alternative projections to extract other modes of spatiotemporal facial variation. For instance, independent components analysis would allow removing the orthogonality constraint, while manifold-learning techniques could derive a non-linear embedding. Such techniques could be used either alongside or instead of PCA.

## Conclusion

We propose a novel PCA-based active appearance model for capturing dimensions of spatial and temporal variation in dynamic facial behaviours. A first-order PCA is used to encode modes of spatial variation in the faces. Representations within this space are then temporally aligned before being submitted to a second-order PCA. Dimensions of this space encode modes of spatiotemporal facial variation. We demonstrate how this technique can be used to produce dynamic anti-caricatures of biological motion patterns in faces, though the general method could be extended to numerous further avenues of research.

## Data Availability Statement

The datasets presented in this study can be found in online repositories. The names of the repository/repositories and accession number(s) can be found below: Open Science Framework (https://osf.io/t6crn/).

## Ethics Statement

The studies involving human participants were reviewed and approved by the Ethics Committee of the School of Psychology at the University of Nottingham (Ethics approval numbers: 717, F1249). The patients/participants provided their written informed consent to participate in this study. Written informed consent was obtained from the individual(s) for the publication of any potentially identifiable images or data included in this article.

## Author Contributions

DW performed the analysis under the supervision of AJ. Both authors conceived and developed the study, contributed to the writing of the manuscript, and approved the final version for submission.

## Author Disclaimer

The views expressed in this article are those of the authors and not necessarily those of the NHS, the NIHR, or the Department of Health and Social Care.

## Conflict of Interest

The authors declare that the research was conducted in the absence of any commercial or financial relationships that could be construed as a potential conflict of interest.

## Publisher’s Note

All claims expressed in this article are solely those of the authors and do not necessarily represent those of their affiliated organizations, or those of the publisher, the editors and the reviewers. Any product that may be evaluated in this article, or claim that may be made by its manufacturer, is not guaranteed or endorsed by the publisher.

## Footnotes

1
https://cloud.google.com/speech-to-text

2
https://opencv.org/

3
https://dynamictimewarping.github.io/

4
https://pavlovia.org/

5
https://github.com/runehaubo/ordinal

## Supplementary Material

Supplementary Video 1 Visualisations of the first five components from the PCA_1_ space for all datasets. Animations illustrate projections between ± 3 standard deviations along each given principal component axis. GN, “Good news”; ISTS, “I’m sorry to say.”

Supplementary Video 2 Back-projection of PCA_1_ timeseries averaged over clips following temporal alignment. To aid visualisation, videos are played at half speed. GN, “Good news”; ISTS, “I’m sorry to say.”

Supplementary Video 3 Visualisations of the first five components from the PCA_2_ space for S1 – “Good news” dataset. Samples were selected at positions between ± 3 standard deviations along a given principal component axis and back-projected to the image space. To aid visualisation, videos are played at half speed.

Supplementary Video 4 As per Supplementary Video 3, for S1 – “I’m sorry to say” dataset.

Supplementary Video 5 As per Supplementary Video 3, for S2 – “Good news” dataset.

Supplementary Video 6 As per Supplementary Video 3, for S2 – “I’m sorry to say” dataset.

Supplementary Video 7 As per Supplementary Video 3, for S3 – “Good news” dataset.

Supplementary Video 8 As per Supplementary Video 3, for S3 – “I’m sorry to say” dataset.

Supplementary Video 9 Dynamic caricatures for an example S1 - “Good news” clip. Multiplication by zero yields the average video sequence, and multiplication by one reproduces the original clip. Intermediate values yield varying levels of anti-caricature, values greater than one yield caricatures, and negative values yield anti-face (anti-)caricatures. To aid visualisation, videos are played at half speed.

Supplementary Video 10 As per Supplementary Video 9, for an example S1 – “I’m sorry to say” clip.

Supplementary Video 11 As per Supplementary Video 9, for an example S2 – “Good news” clip.

Supplementary Video 12 As per Supplementary Video 9, for an example S2 – “I’m sorry to say” clip.

Supplementary Video 13 As per Supplementary Video 9, for an example S3 – “Good news” clip.

Supplementary Video 14 As per Supplementary Video 9, for an example S3 – “I’m sorry to say” clip.
