# Supplementary material for: A PCA-Based Active Appearance Model for Characterising Modes of Spatiotemporal Variation in Dynamic Facial Behaviours
Source: Front Psychol. 2022 May 26;13:880548. doi: 10.3389/fpsyg.2022.880548 (PMC9204357; doi:10.3389/fpsyg.2022.880548)
Supplement: Supplementary file 1 [file Table_1.DOCX]

| ***Supplementary Table 1.*** *List of the ten positive and negative spoken phrases.* | |
| --- | --- |
| Good news … | I’m sorry to say … |
| … your loan has been approved! | … you haven’t got the job. |
| … you’ve got the job! | … we can’t do anything for you. |
| … the vendor has accepted your offer! | … we’ve sold out. |
| … your tests have come back clear! | … the operation didn’t go well. |
| … your application has been accepted! | … we can’t extend you the loan. |
| … your promotion has been agreed! | … we’ve found some serious structural problems. |
| … we won the match! | … your application was denied. |
| … the operation went well! | … we’re going to have let you go. |
| … your car passed its MOT! | … a break-in has been reported at your house. |
| … your bid has been successful! | … someone has been accessing your account. |
